# Improving the utilization of insecticide-treated nets for malaria prevention among pregnant women, lactating mothers and children in Sierra Leone: a commentary

**DOI:** 10.1186/s12936-025-05429-z

**Published:** 2025-06-10

**Authors:** Ronald Carshon-Marsh, Erica Di Ruggiero

**Affiliations:** Dalla Lana School of Public Health, University of Toronto, Toronto, ON M5 T 3M7 Canada

**Keywords:** Malaria in pregnancy, ITN utilization, Pregnant women, Sierra Leone

## Abstract

Malaria in pregnancy poses significant public health challenges with severe consequences for mothers, fetuses, and newborns. Despite the proven efficacy of insecticide-treated nets (ITNs), the coverage rate among pregnant women, lactating mothers and young children in sub-Saharan Africa remains suboptimal. For example, in Sierra Leone, only 52% of pregnant women and 50% of children under five years utilize ITNs. This coverage rate fell short of the national target, in which at least 80% of pregnant women are expected to report sleeping under an ITN. While considerable research has examined ITN access and usage in the general SSA population, focused implementation research on these high-risk groups in Sierra Leone is notably lacking. Addressing this gap is vital for enhancing intervention effectiveness and achieving sustained malaria control. The authors of this commentary recommend that further implementation research is needed to investigate the barriers and enabling factors to ITN adoption and utilization in pregnant women, lactating mothers and children under five years of age. Implementation research is crucial for understanding the gap between ITN access and actual use, enabling the design of effective and equitable interventions to boost utilization rates. Implementation research anchored in frameworks like Reach, Effectiveness, Adoption, Implementation, and Maintenance (RE-AIM) offers a pathway to decode these complexities, ensuring that global strategies resonate with local realities. By centering the voices of pregnant women, lactating mothers, and caregivers as well as addressing structural, cultural, and logistical barriers, Sierra Leone can transform ITN coverage into tangible reductions in malaria morbidity and mortality, advancing equity in its march toward elimination.

## Background

Malaria remains a significant, preventable threat caused by *Plasmodium* parasites, primarily transmitted by infected female *Anopheles* mosquitoes. According to the World Health Organization (WHO), there were an estimated 263 million cases of malaria worldwide in 2023, with approximately 597,000 malaria deaths [1]. The WHO African region is disproportionately affected, accounting for 94% of malaria cases and 95% of malaria deaths, with children accounting for the majority of these fatalities. [1]. It is a major public health problem with heightened risk to the pregnant woman and her fetus. Each year, Malaria in Pregnancy (MIP) is responsible for 20% of stillbirths and 11% of all newborn deaths in sub-Saharan Africa (SSA) [2]. To mitigate these risks, the WHO recommends a three-pronged approach for controlling malaria and its effects during pregnancy: (1) the administration of Intermittent Preventive Treatment during pregnancy using sulfadoxine–pyrimethamine (IPTp-SP) that is quality assured; (2) the widespread distribution and consistent use of Long-Lasting Insecticidal Nets (LLINs) or Insecticide-Treated Nets (ITNs); and (3) effective management of cases by providing prompt, quality diagnosis and effective treatment of malaria infections [3].

Pregnant women, lactating mothers and under-five-year-old children in SSA are particularly vulnerable due to immune system changes, developmental factors, and nutritional challenges that make them more susceptible to malaria. Also, socio-cultural factors (such as low levels of education, cultural beliefs and practices, poverty), poor environmental conditions, limited access to healthcare, and health system challenges further increase their vulnerability. There are many dire consequences of malaria in pregnancy on both the mother and fetus or newborn. Malaria in pregnancy can result in maternal anaemia, placental infection, spontaneous abortion, stillbirth, preterm births, severe malaria, and maternal deaths [4]. For the exposed fetus or newborn, consequences include Intra Uterine Growth Restriction (IUGR), low birth weight, fetal anaemia, prematurity, congenital malaria, and early mortality [4]. In a nationally representative mortality surveillance study conducted in Sierra Leone, stillbirths were high, accounting for 34% of the national mortality total, with a stillbirth rate of 15.6 per 1000 live births [5]. Malaria was the leading cause of death, representing 22% of all deaths in all age groups except neonates [5].

This commentary argues for the need to better understand the implementation barriers and enablers related to a complex public health intervention, which is the adoption and utilization of ITNs in the prevention of malaria in pregnancy, using Sierra Leone as an illustrative case example. ITNs are designed to physically block, repel, or kill vector mosquitoes. This targeted public health intervention aims at preventing and controlling the transmission of malaria. It has wide coverage and, beyond the personal protection it provides, ITNs have a communal effect where community members who do not sleep under ITNs may still be protected if a large proportion of the community uses ITNs [6]. They are cost-effective with a high return on investment in terms of health benefits. They are well integrated into the health system, they are a global health priority and have been proven to reduce malaria morbidity and mortality [3, 4, 6].

While considerable research has examined ITN access and usage in the general SSA population, there is limited research on the factors that influence the adoption and practical implementation of ITNs in pregnant women, children under five years and lactating mothers in Sierra Leone, underscoring the need for focused implementation research studies. Sierra Leone’s unique context demands urgent scrutiny and attention from research.

According to the 2021 Sierra Leone Malaria Indicator Survey (SLMIS) [7], only 50% of children under 5 years and 52% of pregnant women aged 15–49 years slept under an ITN the night before the survey. While some progress has been made over the years in ITN utilization in pregnant women, lactating mothers, and under-five-year-old children, more needs to be done to achieve the target. It is expected that at least 80% of pregnant women should report sleeping under an ITN/LLIN based on the target coverage rate set by the National Malaria Control Programme (NMCP). This gap in ITN utilization points to significant implementation barriers.

Implementation research is crucial for identifying and addressing these barriers, as well as for understanding the enabling factors that promote proper ITN usage. Available studies have focused more on the general population; however, it is important that ITN utilization research focuses on these vulnerable groups since they have some unique socio-cultural, economic, and logistical challenges. Such implementation research is particularly rare in Sierra Leone. Implementation research asks questions such as:

1. What are the barriers affecting the implementation and use of ITNs among pregnant women, lactating mothers, and children under five years in Sierra Leone?
2. What contextual factors enable ITN utilization among these vulnerable populations?

## Main text

### Current evidence on barriers and facilitators of ITN utilization with consideration to the context of Sierra Leone

**Fig. 1:**
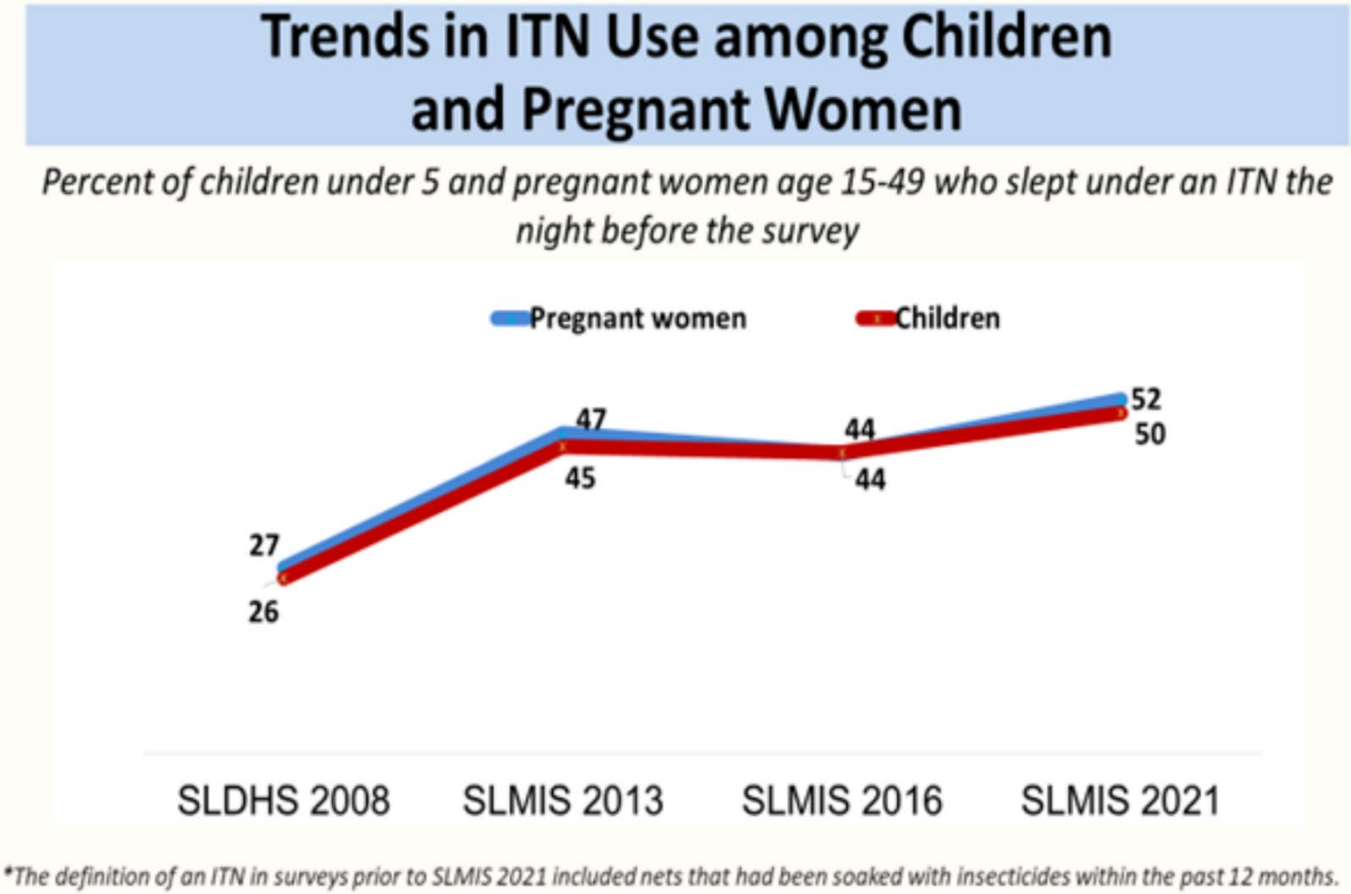
Trends in ITN use among children and pregnant women in Sierra Leone [7]

There has been ample research evidence confirming the efficacy and effectiveness of ITNs in preventing malaria in pregnancy. ITNs have been shown to sharply reduce the malaria burden in countries with high ITN coverage. In a Cochrane review conducted by Gamble et al*.* [8], it was proven that ITNs compared with no nets and untreated nets reduce placental malaria, low birthweight, and stillbirths in the first to fourth pregnancy as well as clinical malaria to some extent. Overall, it has a beneficial impact on pregnancy outcomes in malaria-endemic regions in sub-Saharan Africa. Despite this, the uptake of ITNs remains inconsistent in the region. The graph in Fig. 1 shows the trends in ITN use in pregnant women and under-five-year-old children over years in Sierra Leone. In 2008, only 27% of pregnant women aged between 15 and 49 years slept under an ITN the night before the malaria indicator survey, however, research in 2021 showed that it had improved to 52%, though the national target is yet to be met. The trend is similar for children.

Multiple studies have examined the barriers to ITN use, yet many focus on the general population rather than on high-risk subgroups such as pregnant women, lactating mothers, and young children. In a review by Pulford et al*.* [9], the most widely identified reasons for not using ITNs were discomfort, mainly due to heat and perceived (low) mosquito density. There were social factors reported, such as sleeping elsewhere or not sleeping at all, as well as technical factors, such as not being able to hang the ITN. Other reasons include ITNs being unavailable or misinformation about ITNs. Limited evidence from Sierra Leone provides critical insight into context-specific barriers and enablers [10–12]. A study in eastern Sierra Leone by Gerstl et al. [10] found that 34.2% of 38 households did not hang their ITN because it was not used and still in original packaging, 28.9% used the ITN to wrap mattresses as protection against bedbugs and 28.9% of the respondents said they washed them at the time of interview. Such misuse underscores gaps in education and communication about ITN benefits. Socioeconomic gradients also influence utilization patterns. In a multi-country study involving two districts in Sierra Leone, Babalola et al*.* [11] found that consistent ITN utilization was inversely related to educational status (73.5% among highly educated vs. 81.0% among uneducated individuals) and poorer respondents in the lowest wealth quintile (84.8%) were more consistent with ITN use than those in the highest wealth quintile (71.6%). Ideational factors associated with consistent ITN use in Sierra Leone were perceived self-efficacy to use nets which was the strongest predictor (AOR = 3.246; p < 0.001), then perceived efficacy of nets (AOR = 2.013; p < 0.001), and perceived vulnerability to malaria (AOR = 1.658; p < 0.01) [11].

**Fig. 2:**
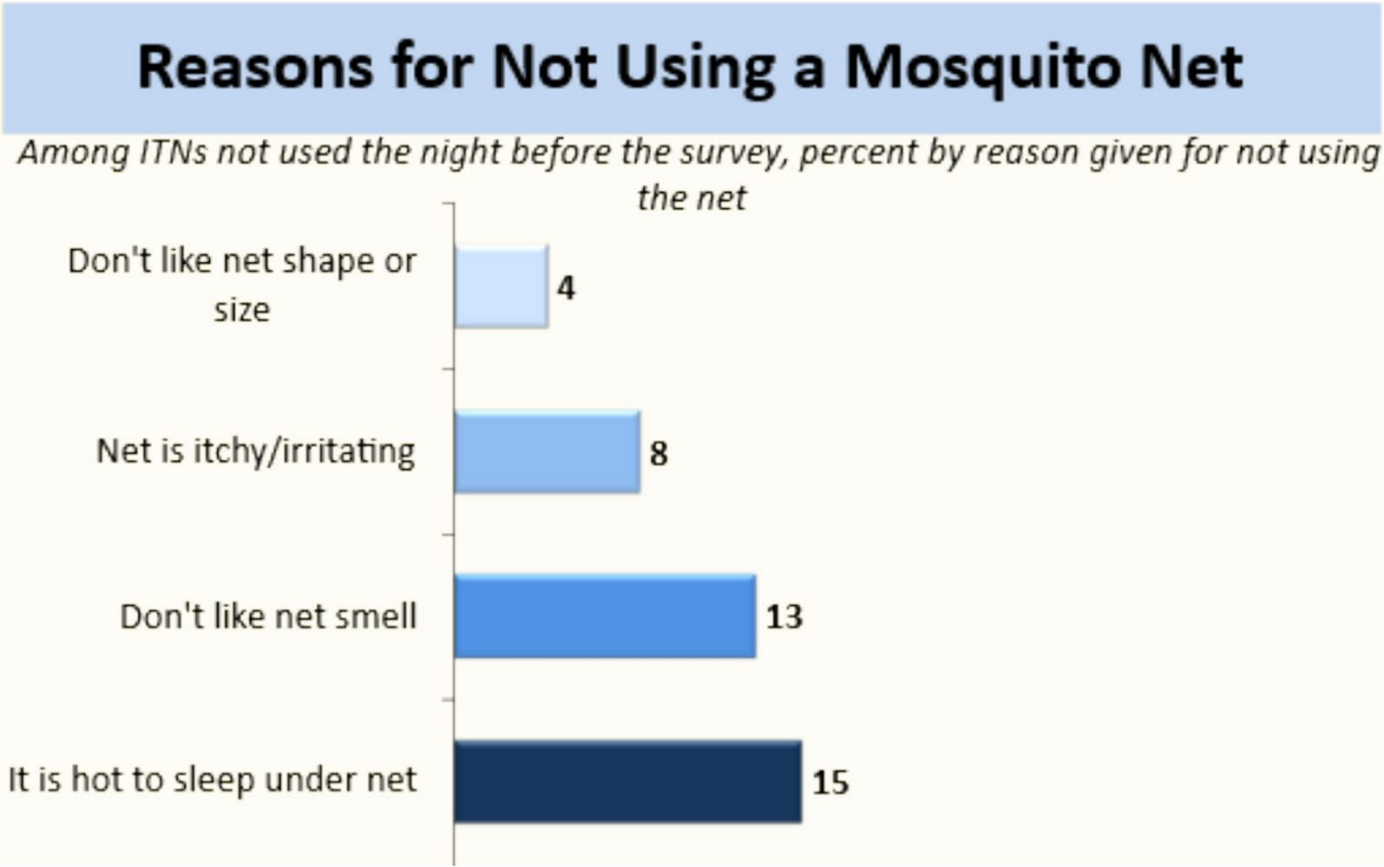
Reasons for not using an ITN among household members in Sierra Leone [7]

During the 2021 Malaria Indicator Survey [7], 40% of ITNs in the households were not used the night before the survey. When asked the reasons for not using the ITNs, most respondents (15%) reported that it was too hot to sleep under a net (Fig. 2).

In a recent study assessing the awareness and effective use of ITNs at Njala University [12], the main facilitating factor of ITN usage was the threat of malaria (46.6%), followed by the accessibility and expense of ITNs (29.8%), and then the comfort provided by ITNs (13.7%). The challenges influencing ITN utilization were identified as malaria risk (43.3%), availability and cost (28.3%), comfort-related issues (12.4%), and 4.6% of the respondents did not know any challenge [12]. These findings underscore the complex interplay between structural, behavioural, and environmental factors shaping ITN use in Sierra Leone. Despite this research, there is a notable gap in implementation research focused specifically on ITN utilization among pregnant women, lactating mothers, and children under five years old, who are disproportionately affected by malaria. These populations face unique barriers, including limited autonomy in decision-making, socio-cultural constraints, and logistical challenges that hinder consistent ITN adoption and usage.

### The imperative for implementation research frameworks

Implementation research offers valuable tools to bridge this knowledge gap. There are various theories, models and frameworks used to guide implementation research. Some commonly used examples are determinant frameworks such as the Consolidated Framework for Implementation Research (CFIR) and the Integrated Promoting Action on Research Implementation in Health Services (i-PARiHS); and the evaluation frameworks, which are the Reach, Effectiveness, Adoption, Implementation, and Maintenance (RE-AIM) and the Implementation Outcomes Taxonomy. The RE – AIM framework is mainly proposed here since the implementation of intervention can be assessed through these five domains at the individual (i.e., end-user), community and organizational (i.e., delivery agent) levels [13]. The RE-AIM framework, which is relatively intuitive, has been widely used over the years in various studies such as planning and evaluating interventions, health promotion and disease management, as well as translating research into practice. It has helped balance the traditional focus of internal validity (within controlled research settings) with external validity (real-world impact and policy implications), but there are challenges implementing it comprehensively, assessing all components, especially in resource-constrained environments [14]. According to Proctor et al*.* [15], adoption is defined as “the intention, initial decision, or action to try or employ an innovation or evidence-based practice”. A pragmatic approach prioritizing adoption and utilization (consistent use) is critical for optimizing ITN delivery and use in this context.

### Distribution channels and acceptability challenges

**Fig. 3:**
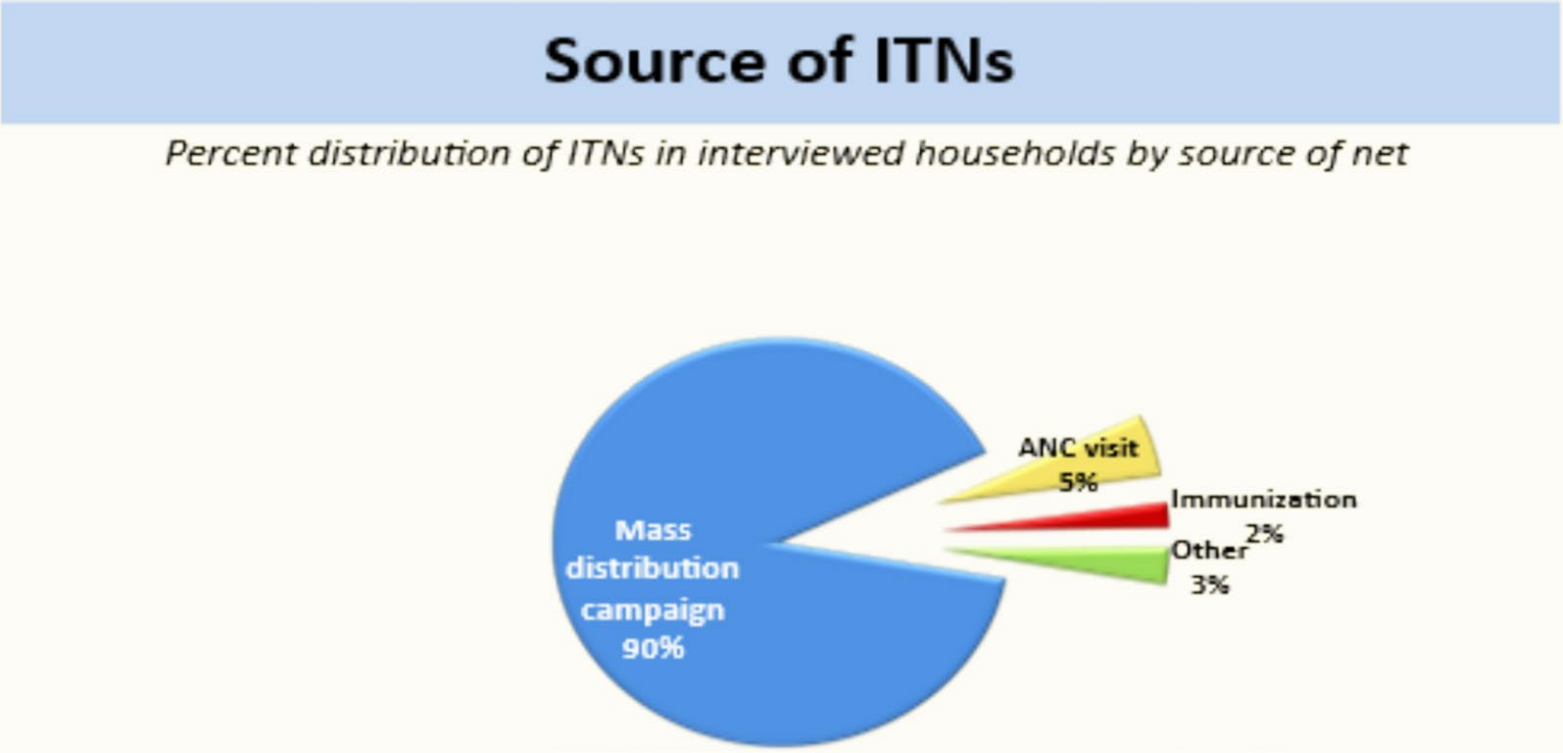
Source of ITNs in Sierra Leone [7]

In Sierra Leone, ITNs are distributed through several channels. They are distributed through antenatal care (ANC) visits, immunization appointments, and triennial mass campaigns led by the National Malaria Control Programme (NMCP). During the mass campaign, one ITN is given to two people in the household and a maximum of three ITNs are given per household for six or more members. Despite 90% of households acquiring ITNs via mass campaigns [7], targeted delivery to pregnant women and lactating mothers remains suboptimal: only 5% receive nets during ANC visits, and 2% during immunization (Fig. 3).

Despite widespread ITN distribution, acceptability issues persist which should be looked into. For example, the ITNs procured and distributed are mostly the hard (polyethylene) type. Eighty-one percent of respondents preferred a soft net material, while 18% preferred a hard material based on the SLMIS survey [7]. These acceptability challenges underscore the need to tailor distribution strategies to user preferences and contextual realities. For example, a process evaluation design would allow for a focus on the targeted ITN deliveries to pregnant women and lactating mothers and provide critical insights into the implementation journey, especially in understanding barriers that hinder ITN utilization and facilitators that promote it, as well as the contextual factors influencing it. It will also help in assessing implementation fidelity, determine how well the ITN distribution, acceptability and utilization targeted programme have adhered to its intended design and protocols. Such evaluation will enable stakeholders to refine strategies, address barriers, allocate resources effectively and enhance the impact of ITNs in malaria prevention for vulnerable populations.

## Recommendations for implementation research and practice

The authors recommend a process implementation evaluation to illuminate the barriers impeding utilization and the contextual enablers supporting ITN use among pregnant women, lactating mothers, and children under five years in Sierra Leone. Implementation research allows for the investigation of contextual influences on health inequities, which have effects at multiple levels, especially the community, institutional, interpersonal, and intrapersonal levels. To improve ITN adoption and utilization among vulnerable groups in Sierra Leone, the authors recommend further implementation research with these priority populations in the Sierra Leone context in mind. For example, a convergent mixed-methods implementation research approach would help to disentangle the interplay of socio-demographic, economic, and health system factors.

The qualitative arm is important for exploring the"how"and"why"behind observed behaviours and system-level dynamics. Additionally, regular, targeted educational campaigns, community engagement and other strategies are recommended to improve awareness and utilization, address misconceptions, and shift behavioural norms among vulnerable groups.

## Conclusions

Preventing malaria in pregnancy is crucial for safeguarding maternal, foetal and neonatal health. While ITNs are a cornerstone of malaria prevention and have demonstrated efficacy, their effectiveness in real-world settings is mediated by numerous implementation factors requiring population group and contextually sensitive implementation research. In Sierra Leone, suboptimal ITN utilization among pregnant women, lactating mothers, and children under five years reflects persistent behavioural, contextual, and systemic barriers that must be urgently addressed. Implementation research, focusing on adoption and utilization outcomes and anchored in frameworks like RE-AIM, offers a pathway to decode these complexities, ensuring that global strategies resonate with local realities. By centering the voices of pregnant women, lactating mothers, and caregivers as well as addressing structural, cultural, and logistical barriers, Sierra Leone can transform ITN coverage into tangible reductions in malaria morbidity and mortality, advancing equity in its march toward elimination. Such evidence is critical not only to reduce malaria-related morbidity and mortality in Sierra Leone but also to inform broader strategies for malaria control across sub-Saharan Africa.

## Data Availability

No datasets were generated or analysed during the current study.

## Abbreviations

CHW: Community health workers
IPTp: Intermittent Preventive Treatment of Malaria during pregnancy
ITN: Insecticide-Treated Nets
LLIN: Long-lasting insecticidal nets
MIP: Malaria in pregnancy
NMCP: National Malaria Control Programme
RE-AIM: Reach, effectiveness, adoption, implementation and maintenance
SP: Sulfadoxine–Pyrimethamine
